# Anaesthetic management for balloon dilation of cor triatriatum dexter in a dog

**DOI:** 10.1186/s13028-015-0119-x

**Published:** 2015-06-10

**Authors:** Valentina De Monte, Francesco Staffieri, Domenico Caivano, Antonello Bufalari

**Affiliations:** Department of Veterinary Medicine, University of Perugia, S. Costanzo Street 4, Perugia, 06126 Italy; Department of Emergency and Organ Transplantation (DETO), Section of Veterinary Clinics and Animal Production, University of Bari, SP per Casamassima km 3, Valenzano, 70010 Italy

**Keywords:** Anaesthesia, Arrhythmia, Balloon dilation, Canine, Cor triatriatum dexter, Puppy

## Abstract

A three-month-old female Rottweiler puppy was referred for intravascular correction of a previously identified cor triatriatum dexter. Echocardiography confirmed the presence of a hyperechoic membrane that divided the right atrium into a cranial and caudal chamber. A foramen in this membrane allowed the blood to flow from the caudal to the cranial chamber. Balloon dilation of the defect under transthoracic echocardiographic guidance was scheduled for the following day. The dog was premedicated with 0.5 μg/kg sufentanil and 0.2 mg/kg midazolam administered intravenously. General anaesthesia was induced with 2 mg/kg propofol and maintained with inhaled isoflurane in oxygen; at the same time, a constant rate infusion of 0.5 μg/kg/h sufentanil was administered by means of an infusion pump. Uneventful ventricular and supraventricular tachyarrhythmias developed during the placement of catheters and balloon dilation. At the end of procedure, when the guide wire and balloon catheter were removed, normal sinus rhythm was observed. To the authors’ knowledge, no previous reports have described the anaesthetic management of a balloon dilation procedure for cor triatriatum dexter in dogs.

## Background

Cor triatriatum dexter (CTD) is a rare congenital defect caused by the failure of the right sinus venosus valve to regress during embryogenesis [1]. The persistent membrane causes the division of the right atrium into a cranial and caudal chamber. Anatomic variations of CTD may be distinguished from the location of the inlet of the coronary sinus, which can enter either the cranial or the caudal chambers. The intra-atrial septum may be imperforate or perforate to varying degrees, resulting in hepatic congestion and ascites due to the mechanical obstruction of the blood flow through the caudal vena cava toward the right atrium [1]. Two techniques have been reported to treat CTD in dogs: surgical membrane resection [2–7] or percutaneous balloon dilation [8, 9].

To the authors’ knowledge, no previous reports have described the anaesthetic management of a balloon dilation procedure for CTD in dogs. This report discusses the anaesthetic protocol used and the impact of balloon dilation on the cardiovascular system.

## Case presentation

A three-month-old female Rottweiler puppy, weighing 11.6 kg, was referred to the cardiology service of the Veterinary Teaching Hospital of Perugia University, Italy for further assessment and treatment of CTD. When presented to the referring veterinarian, the dog had a history of gradual abdominal enlargement over the previous weeks. CDT was diagnosed by transthoracic echocardiography^1^ and medical treatment included furosemide^2^(2 mg/kg orally twice daily), spironolactone^3^ (2 mg/kg orally once daily), and ramipril^4^ (0.25 mg/kg orally once daily). On clinical examination, the dog was bright and alert with a heart rate (HR, beats/min) of 136 and a respiratory rate (RR, breaths/min) of 36, but showed poor body condition (body condition score 3/9) and a markedly enlarged abdomen with a fluid wave. Abdominal ultrasound^5^ revealed severe ascites associated with evident hepatomegaly and hepatic venous congestion. Abdominocentesis revealed modified transudate ascitic fluid (specific gravity 1020, total protein 2.2 g/dL, poor cellularity). Cardiac auscultation revealed no heart murmur or arrhythmia.

Transthoracic echocardiography^5^ showed a thin, hyperechoic membrane that divided the right atrium into a cranial and caudal chamber. The tricuspid valve, cranial vena cava inlet, and right auricle were included in the cranial chamber, whereas the caudal chamber included the caudal vena cava inlet and the coronary sinus. A 4.5 mm opening allowed the blood to flow from the caudal to the cranial chamber. A colour Doppler^5^ examination showed turbulent flow from the caudal to the cranial chamber of the right atrium across the anomalous membrane, with a peak velocity of approximately 2.2 m/sec.

From these findings, CTD was confirmed and correction of the anomalous membrane by a balloon dilation technique was scheduled for the following day. Hematologic parameters had revealed alterations in both blood count^6^ and in biochemical analysis^7^ (Table 1).

**Table 1:** Altered hematologic parameters in a dog undergoing balloon dilation of cor triatriatum dexter

Parameter	Value	Normal range
Hct (%)	28	37–55
Hb (g/dL)	9.84	12–18
RBC (M/μL)	4.68	5.50–8.50
MCV (f/L)	56.5	60–77
RDW (%)	8.6	12–16
TP (g/dL)	3.93	5.5–7.8
Alb (g/dL)	2.5	2.6–3.8
Urea (mg/dL)	49.7	10–45
Ast (U/L)	50	10–40

*Hct* hematocrit, *Hb* haemoglobin, *RBC* red blood cell, *MCV* mean cell volume, *RDW* high red blood cell distribution width, *TP* total protein, *Alb* albumine, *Ast* aspartate transaminase enzymes

Prior to the anaesthetic procedure, the two cephalic veins were aseptically catheterized with a 20 gauge cannula.^8^ Patient preoxygenation (flow-by: 5 L/min of pure oxygen) was allowed before and after premedication. Pre-anaesthesia included consecutive intravenous (IV) administration of 0.5 μg/kg sufentanil^9^ and 0.2 mg/kg midazolam.^10^ Five minutes after premedication, general anaesthesia was induced with 2 mg/kg propofol,^11^ administered as a single, progressive IV bolus. The dog’s trachea was intubated with a 6.5 mm cuffed tube,^12^ which was connected to a non-rebreathing system^13^ (Bain-type). General anaesthesia^14^ was maintained with isoflurane^15^ diluted in oxygen (fresh gas flow: 3 L/min); concurrently, a constant rate infusion (CRI) of 0.5 μg/kg/h sufentanil^9^ and 5 mL/kg/h of lactated Ringer’s solution^16^ were administered IV by infusion pumps.^17^

When an adequate depth of anaesthesia was achieved, the dog was positioned in right lateral recumbency for the cardiovascular corrective procedure. The patient was allowed to spontaneously breathe during the entire anaesthetic procedure and did not required artificial ventilation support.

A 24 gauge cannula^18^ was aseptically inserted into the metacarpal artery to measure direct blood pressure. HR, RR, electrocardiogram (ECG), invasive arterial blood pressure (systolic arterial pressure, diastolic arterial pressure, mean arterial pressure (MAP); mmHg), haemoglobin oxygen saturation (SpO_2_, %), end-tidal carbon dioxide partial pressure (EtCO_2_, mmHg), end-tidal isoflurane concentration (EtIso, %), and rectal temperature (T,°C) were continuously monitored^19^ and recorded every 5 min. During inflation and deflation of the balloon catheter^20^ (BC), the same parameters were recorded every 2 min. The transducer^21^ was zeroed at the level of the manubrium of the sternum.

The interventional procedure included percutaneous access from the right femoral vein by the Seldinger technique [10] and the subsequent positioning of two balloon valvuloplasty catheters^20^ (first 12 mm and then 22 mm) across the intra-atrial membrane, under transthoracic echocardiography guidance^5^. Ten consecutive hand inflations with a 12 mm BC^20^ were performed. The dilation procedure was then repeated by using a 22 mm BC^20^.

**Fig. 1:**
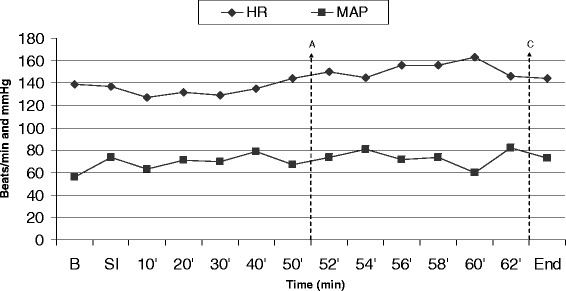
Heart rate (HR, beats/min) and mean arterial pressure (MAP, mmHg) values of the dog undergoing balloon dilation for cor triatriatum dexter at Baseline (**b**, 15 min before the skin incision), at Skin incision (**si**) and every 10 minutes until the start of the balloon inflation-deflation (**a**), when the same parameters are reported every 2 minutes until the end of the procedure (**c**) (End, 15 min after the end of the cardiovascular procedure)

**Fig. 2:**
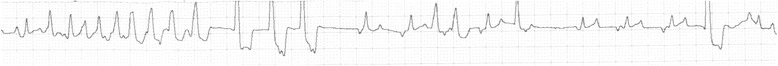
Electrocardiographic findings in a dog with CTD during the 3^d^ balloon inflation (22 mm balloon catheter). The trace reveals atrial (QRS complex similar to a sinus complex), ventricular (wide QRS complex followed by a large T wave) and junctional beats (QRS complex similar to a sinus complex and a negative P wave). The atrial beats are followed by ventricular beats, and junctional beats occurred when the balloon slipped distally against the tricuspid valve. Lead II tracing; Speed 25 mm/s; amplitude 10 mm/mV

**Fig. 3:**
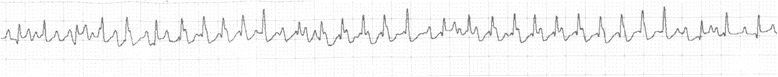
Electrocardiographic findings in a dog with CTD during the last balloon inflation (22 mm balloon catheter). The trace shows a non-sustained supraventricular tachycardia (duration 6 s) with a rate of 250 beats/min. Lead II tracing; Speed 25 mm/s; amplitude 10 mm/mV

The HR from the beginning to the end of the anaesthesia ranged from 126 to 163 and the MAP from 62 to 82 (Fig. 1). Table 2 shows the values of RR, SpO_2_, EtCO_2_, EtIso, and T. During the interventional procedure, several arrhythmias were noted and recorded (ventricular and supraventricular tachyarrhythmias). Transient supraventricular extrasystoles were observed during the placement of the guide wire^22^ and BC^20^, but spontaneously resolved following the withdrawal of the dilation instrumentations from the atrial wall. During balloon inflations (22 mm BC^20^), ventricular and junctional beats (Fig. 2) and non-sustained supraventricular tachycardia (Fig. 3) occurred, with spontaneous conversion to sinus rhythm after the end of the dilation procedure. These arrhythmias did not show any haemodynamic impact on the arterial pressure.

**Table 2:** Physiological parameters for a balloon dilation of cor triatriatum dexter in a dog under general anaesthesia

	Baseline	b-CBI C (range)	CBI (range)	End
RR (breaths/min)	26	22–30	23–27	12
SpO_2_ (%)	95	92–100	94–95	98
EtCO_2_ (mmHg)	38	37–41	39–45	37
EtIso (%)	1.2	1.1–1.2	1.1–1.2	1.3
T (°C)	36.5	35.9–36.5	35.6–35.9	35.2

*Baseline* 15 min before the skin incision, *End* 15 min after the end of the procedure, *CB* catheter balloon, *b-CBI* form Baseline to the beginning of CB inflation, *CBI* during CB inflation, *RR* respiratory rate, *SpO*_*2*_ haemoglobin oxygen saturation, *EtCO*_*2*_ end-tidal carbon dioxide partial pressure, *EtIso* end-tidal isoflurane concentration, *T* rectal temperature

Table 3 provides the total anaesthesia and cardiovascular procedure time, the total inflation-deflation time of the BC^20^, the number of BC^20^ inflations, and the total dose and CRI time of sufentanil^9^.

**Table 3:** Anaesthetic protocol and details of cardiovascular procedure for a balloon dilation of cor triatriatum dexter in a dog

Premedication	Sufentanil (0.5 μg/kg) + Midazolam (0.2 mg/kg)
Induction	Propofol (2 mg/kg)
Maintenance	Isoflurane in O_2_ + Sufentanil CRI (0.5 μg/kg/h)
Total anaesthesia time	117 min
Total cardiovascular procedure time	62 min
Total time of the inflation-deflation of the BC	12 min
Number of balloon inflations	20; 10 (12 mm BC) + 10 (22 mm BC)
Total sufentanil CRI time	97 min
Total dose of sufentanil	15.1 μg

*BC* balloon catheter, *CRI* constant rate infusion

The dog was extubated 9 min after the end of the anaesthesia and was able to stand after 14 min. The recovery was uneventful and the patient was hospitalized post-operatively for two days.

## Conclusions

Although a definitive conclusion cannot be made from a single case, the balanced anaesthesia used for the three-month-old puppy described in this report assured good patient haemodynamic stability for the safeness of this minimal invasive balloon dilation technique. The side effects that occurred, such as supraventricular extrasystoles, supraventricular tachycardia, and ventricular and junctional beats, did not have any haemodynamic impact and disappeared as soon as the wires were removed from the heart. Antiarrhythmic therapy was therefore not required.

During an interventional procedure, anaesthesia should ensure hypnosis, analgesia, and haemodynamic stability; titration should be easy and reliable in order to cope with rapidly changing loading conditions and intense, but short-lived, haemodynamic disturbances [11]. The anaesthetic drugs should not interfere with the electrophysiological processes in the cardiac conduction system and, above all, anaesthetic recovery should be fast and free of side effects [11]. Furthermore, puppies under anaesthesia are more likely to develop hypotension, hypothermia, hypoglycaemia, and hypoxia [12, 13]. For these reasons, management of the anaesthesia for a paediatric dog undergoing an interventional procedure is extremely critical. In addition to these fundamental principles, anaesthetists dealing with a patient with CTD must also follow the haemodynamic principles used to manage patients with tricuspid stenosis: avoid tachycardia, avoid hypotension by maintaining euvolemia, and avoid increases in pulmonary vascular resistance [14].

The veterinary literature lacks information about the anaesthesia strategy, the interactions of drugs and the repercussions for the patient, and the possible cardiovascular impact of the drugs chosen in dogs affected by CTD who are undergoing balloon dilation of the defect. Some reports indicate only which anaesthetic drugs were used [8, 9, 15, 16]. In our case, the dog was sedated with a combination of a potent opioid (sufentanil) and midazolam in order to preserve the cardiovascular function of the patient [17, 18]. Premedication is a crucial phase of anaesthesia for a veterinary patient; it can calm the animal and decrease the amount of induction and maintenance drugs that are needed [19]. Mild sedation is desirable to prevent excitement during anaesthetic induction and to avoid excess sympathetic tone, which can cause cardiac arrhythmia or ventricular fibrillation [20, 21].

Opioids are the preferred agents in critically ill patients because of the minimal effect of these agents on cardiac output (CO), systemic blood pressure, and oxygen delivery. They also provide analgesic and sedative effects that can be reversed with naloxone, if necessary [22]. Generally speaking, however, at high dosages, they cause bradycardia mediated via a vagal mechanism [21]: a slowing of HR in puppies results in decreased CO due to the limited capacity of the heart to increase cardiac stroke volume [13]. These side effects must always be carefully weighed against their benefits, particularly in terms of sedation and analgesia. Atropine, an anticholinergic drug used to decrease respiratory tract secretions and to treat bradyarrhythmias, was not included in our protocol because of its intrinsic arrhythmogenic potential [23, 24] and the probable tachyarrhythmias that the procedure itself could have caused [9, 15, 16].

Benzodiazepines such as midazolam and diazepam are unreliable anxiolytics when given alone, unless the animal has central nervous system depression; however, they are able to provide improved sedation when given in combination with opioids and dissociatives [22]. Furthermore, young puppies, unlike adult dogs, are more susceptible to the sedative effects of benzodiazepines.

Acepromazine, a phenothiazine derivate, is largely used as a sedative in dogs, but its tendency to cause mild hypotension by blocking α-1 receptors and its long half-life made it unsuitable for use in our case. Alpha-2 agonists (e.g. dexmedetomidine, medetomidine) are excellent sedatives and anxiolytics, but significantly affect cardiovascular function. The main negative cardiovascular effects of these drugs include bradycardia with possible bradyarrhythmias (atrioventricular heart block and ventricular escape rhythm) and a dramatic reduction in CO caused by reduced central sympathetic tone. These molecules also cause an increase in systemic vascular resistance (peripheral action on α-2 receptors) and hypertension, which induces a reflex baroreceptor-mediated physiologic bradycardia and marked reduction in CO, further perpetuated by the central effects of sedation and reduced sympathetic tone [17, 25]. Healthy paediatric patients have limited stroke volume and cardiac reserve, and CO is dependent on HR [13]; therefore, this class of drugs, which causes bradycardia and a decrease in CO, was not considered for the anaesthetic management of this puppy. Furthermore, the cardiovascular procedure itself could cause arrhythmias, and so these drugs were excluded from the protocol.

Ideally, after the clinical effect of premedication, a slow transition to general anaesthesia should occur to allow time for the cardiovascular and nervous system to appropriately respond to and accommodate the medications [26]. Propofol is a hypnotic agent, which, because of its multiple routes of elimination and its smooth induction, especially for recovery, is most commonly used for paediatric patients to induce general anaesthesia [21, 27, 28]. Cardiovascular changes induced by propofol administration consist of a slight decrease in arterial blood pressures (systolic, mean, diastolic) without a compensatory increase in HR, and so the dose of propofol should always be carefully titrated against the needs and responses of individual patients, as their anaesthetic requirements vary considerably [28]. In our case, the dog required a low dose of propofol for the induction of general anaesthesia. Propofol binds strongly to serum protein (97–98 %); hence, in this specific puppy, hypoproteinaemia (hypoalbuminaemia and hypohaemoglobinemia) resulted in an increase of the unbound drug in the circulation (i.e. the active part of the drug), which may have been further increased by the strong protein binding of the other drugs used for premedication. Thus, we can assume that the poor clinical condition of the patient contributed to a lower than expected volume of distribution and, therefore, in a higher serum drug concentration. These possible contributing factors, added to the effects of premedication, probably justify the low dose of propofol used for induction of general anaesthesia.

All volatile inhalation anaesthetics cause dose-dependent and drug-specific changes in cardiovascular performance: decreased arterial blood pressure, stroke volume, and peripheral vascular resistance. The newer volatile anaesthetics tend to preserve CO at clinically useful concentrations [29, 30]. A major advantage of these newer agents (sevoflurane, desflurane) is their superior pharmacokinetic properties, including low solubility in blood and tissues [31], resulting in a faster induction and recovery and improved control of the depth of anaesthesia [32]. However, recent veterinary literature lacks clear evidence of better clinical performance for sevoflurane and desflurane, compared with isoflurane, in non-critical dogs and cats. Furthermore, both isoflurane and sevoflurane are used in cardiac surgery in humans, resulting in a similar cardioprotective effect [33, 34].

Sufentanil decreases the minimum alveolar concentration (MAC) of volatile anaesthetics [35, 36]. In our case, isoflurane end tidal concentration was maintained mostly below 1 MAC (1 MAC = 1.28 vol.%), resulting in good haemodynamic stability (Table 2). Moreover, sufentanil and other potent opioids can have a cardioprotective and antiarrhythmic effect mediated by diverse intracellular signalling pathways and indirect effects [37, 38]. Indeed, this specific μ-opioid receptor agonist is largely used for cardiac surgery anaesthesia in children [39, 40]. On the other hand, the possible occurrence of bradycardia due to vagal stimulation is an undesirable event in a puppy because of the negative effect that bradycardia may have on CO. Little information has been reported in the literature about pain during minimally invasive vascular procedures. Portmans asserts that the inflation of the balloon (e.g. dilation of a stenotic valve) can cause pain, which is essentially acute and intense, but short-lived [11]. Thus, the reasons for choosing the potent opioid sufentanil for the management of anaesthesia include its halogen-sparing effects, its cardioprotective and analgesia effects during inflation of the balloon in the atrium, and the possibility of controlling undesirable bradycardia with the administration of atropine.

The puppy in this report, despite having hypoproteinaemia, an obstructed venous return, and an extremely tense abdomen, did not require any drugs to support cardiovascular function, because the physiological parameters were within normal ranges throughout the entire procedure. Hypoproteinaemia usually causes an increase in the active, unbound plasma concentration of drugs, and thus patients with this condition should be treated cautiously, as they are more prone to develop side effects from a relative overdose [41]. Hypoproteinaemia can also affect oncotic pressure, making the dog more prone to hypovolemia and hypotension [42].

The use of spontaneous ventilation in our patient was suggested for two main reasons: 1) Patients undergoing minimally invasive procedures usually do not require a surgical plane of anaesthesia and therefore most of the time do not easily adapt to the ventilator unless more anaesthetic drug or a neuromuscular blocker is administered; and 2) positive pressure ventilation may interfere with cardiovascular function, especially in haemodynamically compromised patients, whereas spontaneous ventilation may better preserve venous return because of negative intrathoracic pressure during inspiration [43]. In any case, positive pressure ventilation should always be available and ready to be used in case the patient’s condition deteriorates.

During CTD dilation, atrial fibrillation, atrial flutter, and ventricular and supraventricular tachyarrhythmias have been reported in dogs [9, 15, 16]. One minute of cardiac arrest, successfully treated with internal cardiac massage and adrenaline injection, was described during the surgical treatment of CTD in a dog [7]. Although tachyarrhythmias appear to be most frequent, many types of arrhythmias may occur (atrioventricular block, tachy-bradyarrhythmias, cardiac arrest) [16, 44–46], and so the authors considered it more appropriate to administer the correct antiarrhythmic drug when needed, rather than administering it preventively.

In our case, supraventricular extrasystoles observed during the placement of the guide wire or balloon catheters were likely due to the contact of the catheter tip with the atrial wall. Supraventricular tachycardia occurred during balloon inflation as a consequence of atrial membrane and wall stretching. Ventricular and junctional beats were recorded when the balloon slipped distally to the membrane against the tricuspid valve during inflation. The fact that arrhythmias disappeared and baseline cardiovascular and respiratory parameters resumed at the end of the procedure suggests that these side effects were caused by the mechanical action of the catheters and guide wire on the cardiac structures (Table 3). Despite the numerous arrhythmias, the invasive blood pressure values did not show any major variations during balloon inflation (Fig. 1), probably because each individual dilation was brief (3–5 s) and because a 20- to 30-s pause was guaranteed between inflations in order to allow the blood to flow through the cardiac chambers.

In our experience, perfect interaction between cardiologist and anesthesiologist is the basis of patient management; on request by the anesthesiologist, displacement of the intracardiac catheter by even a few millimeters led to total interruption of the arrhythmias.

Although the arrhythmias recorded in our case report were benign and might be explained by the contact of the catheters and guide wire on the endocardial wall, it must be emphasized that the supraventricular and ventricular arrhythmias may place the patient at higher risk for development of malignant arrhythmias. Therefore, anti-arrhythmic drugs (e.g. lidocaine, esmolol, atropine) and a defibrillator must be available during the interventional procedure. Lidocaine IV via bolus or CRI has been used to provide perioperative antiarrhythmic effects and has been assessed as advantageous for dogs undergoing balloon valvuloplasty for pulmonic stenosis [47].

Our findings are in complete agreement with those described in the literature regarding the occurrence of arrhythmias and their spontaneous resolution following withdrawal of the catheters. The protocol described in this case report resulted in good management of anaesthesia and analgesia, as well as a fast, smooth, and uneventful recovery. Although in particular cases, it is highly recommended to tailor the anaesthetic protocol to the individual patient’s clinical condition, the protocol described in this case can be considered a valid basic approach to be adapted as necessary; moreover, considerable attention should be given to arrhythmias that develop during the procedure to provide timely intervention.

## Abbreviations

BC: Balloon catheter
CRI: Constant rate infusion
CTD: Cor triatriatum dexter
DAP: Diastolic arterial pressure
ECG: Electrocardiogram
EtCO_2_: End-tidal carbon dioxide partial pressure
EtIso: End-tidal isoflurane concentration
HR: Heart rate
MAP: Mean arterial pressure
RR: Respiratory rate
SAP: Systolic arterial pressure
SpO_2_,: Haemoglobin oxygen saturation
T: Rectal temperature

## Footnotes

1 MyLab 30 Vet, Esaote, Genova, Italy

2 Diuren; Teknopharma S.p.A., Torino, Italy

3 Prilactone; Ceva Vetem S.p.A., Monza e della Brianza, Italy

4 Vasotop P; MSD Animal Health S.r.l., Latina, Italy

5 MyLab™30 Vet Gold; Esaote, Genova, Italy

6 Cell-Dyn 3700; Abbott, Roma, Italy

7 Liasys; Assel S.r.l., Latina, Italy

8 Delta ven® 1 G 22; Delta Med S.p.A., Mantova, Italy

9 Sufentanil-hameln; Hospira Srl, Napoli, Italy

10 Midazolam I.B.I.; Hospira, Lake Forest, IL, USA

11 Proposure 1 %; Merial S.p.A., Milano, Italy

12 Rüsch® 6.5 mm endotracheal cuffed tube; Teleflex Medical S.r.l., Monza e della Brianza, Italy

13 Infant T-piece anaesthetic breathing system; Intersurgical S.p.A., Modena, Italy

14 Excel 210; Ohmeda, Madison, WI, USA

15 Isoflurane-Vet; Merial, Milano, Italy

16 Ringer Lattato; S.A.L.F S.p.A., Bergamo, Italy

17 SK500II sirynge pump; Foschi S.r.l., Roma, Italy

18 Delta ven® 1 G 24; Delta Med SPA, Mantova, Italy

19 Multiparameter Monitor HB100; Foschi, Roma, Italy

20 Tyshak II® balloon valvuloplasty catheter; Infiniti Medical, LLC™, West Hollywood, CA, USA

21 DPT-6000; Codan srl, Modena, Italy

22 Rosen Wire; Infiniti Medical, LLC™, West Hollywood, CA, USA

## References

[1] Kittleson MD, Kienle RD, Kittleson MD, Kienle RD (1998). Other congenital cardiovascular abnormalities. Small animal cardiovascular medicine.

[2] Tobias AH, Thomas WP, Kittleson MD, Komtebedde J (1993). Cor triatriatum dexter in two dogs. J Am Vet Med Assoc.

[3] Kaufman AC, Swalec KM, Mahaffey MB (1994). Surgical correction of cor triatriatum dexter in a puppy. J Am Anim Hosp Assoc.

[4] Mitten RW, Edwards GA, Rishniw M (2001). Diagnosis and management of cor triatriatum dexter in a Pyrenean mountain dog and an Akita Inu. Aust Vet J.

[5] Hoffmann DE, Tobias AH (2003). What is your diagnosis? Cor triatriatum Dexter. J Am Vet Med Assoc.

[6] Tanaka R, Hoshi K, Shimizu M, Hirao H, Akiyama M, Kobayashi M (2003). Surgical correction of cor triatriatum dexter in a dog under extracorporeal circulation. J Small Anim Pract.

[7] Chanoit G, Bublot I, Viguier E (2009). Transient tricuspid valve regurgitation following surgical treatment of cortriatriatum dexter in a dog. J Small Anim Pract.

[8] Adin DB, Thomas WP (1999). Balloon dilation of cor triatriatum dexter in a dog. J Vet Intern Med.

[9] Johnson MS, Martin M, Giovanni JVD, Boswood A, Swift S (2004). Management of cor triatriatum dexter by balloon dilatation in three dogs. J Small Anim Pract.

[10] Estrada A, Moïse NS, Renaud-Farrell S (2005). When, how and why to perform a double ballooning technique for dogs with valvular pulmonic stenosis. J Vet Cardiol.

[11] Poortmans G (2004). Anaesthesia for children with congenital heart disease undergoing diagnostic and interventional procedures. Curr Opin Anaesthesiol.

[12] Grandy JL, Dunlop CI (1991). Anesthesia of pups and kittens. J Am Anim Hosp Assoc.

[13] Thurmon JC, Tranquilli WJ, Benson GJ, Thurmon JC, Tranquilli WJ, Benson GJ (1996). Neonatal and geriatric patients. Lumb & jones veterinary anesthesia.

[14] Wallace A, Stoelting RK, Miller RD (2007). Cardiovascular disease. Basics of Anesthesia.

[15] Lòpez-Alvarez J, Dukes-McEwan J, Martin MW, Killick D, Fonfara S, Fraser McConnell J (2011). Balloon dilation of an imperforate cor triatriatum Dexter in a golden retriever with concurrent double-chambered right ventricle and subsequent evaluation by cardiac magnetic resonance imaging. J Vet Cardiol.

[16] Leblanc N, Defrancesco TC, Adams AK, Atkins CE, Tou SP, Fudge JC (2012). Cutting balloon catheterization for interventional treatment of cor triatriatum dexter: 2 cases. J Vet Cardiol.

[17] Congdon JM, Snyder LBC, Johnson RA (2015). Cardiovascular disease. Canine and feline anesthesia and co-existing disease.

[18] Fávaroda Cunha A, Snyder LBC, Johnson RA (2015). Neonatal, pediatric, and geriatric concerns. Canine and feline anesthesia and co-existing disease.

[19] Quandt J (2013). Analgesia, anesthesia, and chemical restraint in the emergent small animal patient. Vet Clin North Am Small Anim Pract.

[20] Thurmon JC, Tranquilli WJ, Benson GJ, Thurmon JC, Tranquilli WJ, Benson GJ (1996). Preanesthetics and anesthetic adjuncts. Lumb & Jones Veterinary Anesthesia.

[21] Hall LW, Clarke KW, Trim CM, Hall LW, Clarke KW, Trim CMWB (2001). Principles of sedation, analgesia and premedication. Veterinary anaesthesia.

[22] Macintire DK, Drobatz KJ, Haskins SC, Saxon WD, Macintire DK, Drobatz KJ, Haskins SC, Saxon WD (2005). Anesthetic protocols for short procedures. Manual of small animal emergency and critical care.

[23] Muir WW (1978). Effects of atropine on cardiac rate and rhythm in dogs. J Am Vet Med Assoc.

[24] Popilskis SJ, Lee DR, Elmore DB, Fish RE, Brown MJ, Danneman PJ, Karas AZ (2008). Anesthesia and analgesia in nonhuman primates. Anesthesia and analgesia in laboratory animals.

[25] Sinclair MD (2003). A review of the physiological effects of alpha2-agonists related to the clinical use of medetomidine in small animal practice. Can Vet J.

[26] Trim CM, Paddleford RR (1999). Anesthetic considerations and complications. Manual of small animal anesthesia.

[27] Grubb TL, Carroll GL (2008). Anesthesia for patients with special concerns. Small animal anesthesia and analgesia.

[28] Short CE, Bufalari A (1999). Propofol anesthesia. Vet Clin North Am Small Anim Pract.

[29] Merin RG, Bernard JM, Doursout MF, Cohen M, Chelly JE (1991). Comparison of the effects of isoflurane and desflurane on cardiovascular dynamics and regional blood flow in the chronically instrumented dog. Anesthesiology.

[30] Steffey EP, Thurmon JC, Tranquilli WJ, Benson GJ (1996). Inhalation anesthetics. Lumb & jones veterinary anesthesia.

[31] Eger EI, Shafer SL (2005). The complexity of recovery from anesthesia. J Clin Anesth.

[32] Young CJ, Apfelbaum JL (1995). Inhalational anesthetics: desflurane and sevoflurane. J Clin Anesth.

[33] Lee MC, Chen CH, Kuo MC, Kang PL, Lo A, Liu K (2006). Isoflurane preconditioning-induced cardio-protection in patients undergoing coronary artery bypass grafting. Eur J Anaesthesiol.

[34] Hemmerling T, Olivier JF, Le N, Prieto I, Bracco D (2008). Myocardial protection by isoflurane vs. sevoflurane in ultra-fast-track anaesthesia for off-pump aortocoronary bypass grafting. Eur J Anaesthesiol.

[35] Brunner MD, Braithwaite P, Jhaveri R, McEwan AI, Goodman DK, Smith LR (1994). MAC reduction of isoflurane by sufentanil. Br J Anaesth.

[36] Bufalari A, Di Meo A, Nannarone S, Padua S, Adami C (2007). Fentanyl or sufentanil continuous infusion during isoflurane anaesthesia in dogs: clinical experiences. Vet Res Commun.

[37] Lishmanov YB, Maslov LN (2004). Opioid receptors and heart resistance to arrhythmogenic factors. Bull Exp Biol Med.

[38] Luna-Ortiz P, Zarco-Olvera G, Ramírez-Ortega M, Tenorio-López FA, Gutiérrez A, Martínez-Rosas M (2009). Antiarrhythmic and cardioprotective effects of remifentanil in anesthetized dogs. Arch Cardiol Mex.

[39] Glenski JA, Friesen RH, Hassanein RS, Henry DB (1988). Comparison of the hemodynamic and echocardiographic effects of sufentanil, fentanyl, isoflurane, and halothane for pediatric cardiovascular surgery. J Cardiothorac Anesth.

[40] Lundeberg S, Roelofse JA (2011). Aspects of pharmacokinetics and pharmacodynamics of sufentanil in pediatric practice. Paediatr Anaesth.

[41] Thurmon JC, Tranquilli WJ, Benson GJ, Thurmon JC, Tranquilli WJ, Benson GJ (1996). Consideration for general anesthesia. Lumb & Jones Veterinary Anesthesia.

[42] de Brito Galvao JF, Center SA, DiBartola SP (2012). Fluid, electrolyte, and acid–base disturbances in liver disease. Fluid, electrolyte, and acid–base disorders in small animal practice.

[43] Shekerdemian L, Bohn D (1999). Cardiovascular effects of mechanical ventilation. Arch Dis Child.

[44] Steinberg C, Levin AR, Engle MA (1992). Transient complete heart block following percutaneous balloon pulmonary valvuloplasty: treatment with systemic corticosteroids. Pediatr Cardiol.

[45] Gupta PK, Haft JI (1972). Complete heart block complicating cardiac catheterization. Chest.

[46] Sun F, Usón J, Crisóstomo V, Maynar M (2005). Interventional cardiovascular techniques in small animal practice-diagnostic angiography and balloon valvuloplasty. J Am Vet Med Assoc.

[47] Ramos RV, Monteiro-Steagall BP, Steagall PV (2014). Management and complications of anaesthesia during balloon valvuloplasty for pulmonic stenosis in dogs: 39 cases (2000–2012). J Small Anim Pract.

